# Nexus: A versatile console for advanced low‐field MRI

**DOI:** 10.1002/mrm.30406

**Published:** 2025-01-27

**Authors:** David Schote, Berk Silemek, Thomas O'Reilly, Frank Seifert, Jan‐Lukas Assmy, Christoph Kolbitsch, Andrew G. Webb, Lukas Winter

**Affiliations:** ^1^ Department 8.1 ‐ Biomedical Magnetic Resonance Physikalisch‐Technische Bundesanstalt (PTB) Braunschweig and Berlin Germany; ^2^ Department of Radiology Leiden University Medical Center Leiden Netherlands

**Keywords:** EMI mitigation, low‐field MRI, MR console, open source, sensors

## Abstract

**Purpose:**

To develop a low‐cost, high‐performance, versatile, open‐source console for low‐field MRI applications that can integrate a multitude of different auxiliary sensors.

**Methods:**

A new MR console was realized with four transmission and eight reception channels. The interface cards for signal transmission and reception are installed in PCI Express slots, allowing console integration in a commercial PC rack. Following standards developed by the MRI community, we implemented an open‐source console software package with native Pulseq and ISMRM raw data support. It is implemented in *Python* to allow easy customization and provide the flexible use of a freely configurable number of transmit and receive channels. We benchmarked the system by comparing the imaging quality with a state‐of‐the‐art reference system. Different examples of how auxiliary sensors, connected via additional channels, can improve imaging are demonstrated.

**Results:**

Using a three‐dimensional turbo spin‐echo sequence, image quality of proton density–weighted and T_2_‐weighted images in the brain of a healthy volunteer obtained by the proposed Nexus console matches closely to a commonly applied commercial system. The use of additional receive channels was demonstrated for system monitoring (radiofrequency pulses and gradient currents), electromagnetic interference detection, and temperature and B_0_ field monitoring. Based on these measurements, system calibrations and electromagnetic interference–mitigation techniques were applied to improve image quality.

**Conclusion:**

Our console offers high versatility in terms of data acquisition, is freely configurable, adheres to open‐source data standards, and is easy to customize. It yields a similar image quality compared with a commercially available reference system yet is substantially lower cost and open source.

## 1 INTRODUCTION

MRI technology has undergone considerable advances in recent years, with a growing emphasis on enhancing accessibility and affordability through portable point‐of‐care low‐field scanners.^1^, ^2^, ^3^, ^4^, ^5^, ^6^, ^7^, ^8^ Target imaging regions of these systems with B_0_ field strengths of about 50 mT include the brain,^9^, ^10^ extremities,^11^, ^12^ and, most recently, even whole‐body imaging.^13^ The compact design of the scanners enables a wide range of applications,^14^ including use in intensive care units,^15^, ^16^ at the bedside,^17^, ^18^, ^19^ in ambulances,^20^ or at patients' homes.^21^ These systems are all based on permanent magnet technology, with both academic and commercial systems available, and designs based on C‐shaped, H‐shaped, and Halbach‐based geometries.

Open‐source hardware and software designs have emerged as key strategies to address accessibility to these portable, low‐cost systems.^22^, ^23^ Examples include the OSI^2^ ONE^24^ scanner, designed for imaging head and extremities, or the MRI4ALL^25^ and OCRA^26^ tabletop scanner intended for educational use. These open‐source designs enable innovation, customization to local needs, and cost‐effective implementation (material costs < 50 000 €) in low‐resource settings, making them in many ways more accessible. Beyond affordability, standardized assembly protocols, sustainable global sourcing, and knowledge transfer for construction, operation, and maintenance promise to further democratize these technologies in the mid to long term.

Portable low‐field MRI scanners face several challenges in optimizing and maintaining image quality.^27^, ^28^ The signal‐to‐noise ratio (SNR) is inherently lower compared with higher‐field systems, and operating outside controlled clinical environments further degrades SNR due to additional noise from electromagnetic interference.^29^ Compact low‐field scanners with permanent magnets have larger B_0_ field inhomogeneities (>100 ppm) and are prone to B_0_ field drifts due to the temperature‐dependent field of the magnets, causing image distortions.^30^, ^31^ These issues can be mitigated by additional sensors or more powerful image reconstruction methods. Physics‐informed deep learning^32^ and hardware solutions like B_0_ field probes and active shimming coils^33^ have shown promising results in improving B_0_ field homogeneity. For the detection and mitigation of electromagnetic interference (EMI), the signals acquired by additional radiofrequency (RF)–sensing coils positioned around the system can be used to eliminate EMI by computational methods.^34^ A deep learning–based approach^35^ has been shown to further reduce EMI artifacts by 42% compared with analytical methods.^36^ To enhance the image quality by these techniques, specialized hardware interfaces with the MRI system and significant computational resources for efficient image reconstruction are required.

The MR console, which controls signal generation, acquisition, image reconstruction, and integration of sensor information, is integral to a point‐of‐care system and can be the most expensive component in such a system. Currently available consoles are, however, generally limited in technical specifications, accessibility and/or affordability, preventing the implementation of advanced low‐field techniques in low‐resource settings. Consoles with proprietary software pose constraints in the customizability and the compatibility to existing open‐source software for pulse sequence development^37^, ^38^ or open data formats,^39^ which enable direct access to existing reconstruction algorithms.^40^ The scarcity of information and limited access impede technical innovations for this critical component, resulting in high costs and redundant engineering efforts.^23^ Open‐source software frameworks for the console exist, which can be used with proprietary software defined radios,^41^ but are limited to two transmit and receive channels. The current lack of on‐board computational power prevents the implementation of advanced imaging techniques, which require real‐time feedback from additional sensors or dynamically optimized imaging parameters. Channeling data of many different sensors within the console enables self‐calibration and correction techniques, enhancing the overall system reliability and simplifies the use. This will ultimately reduce the amount of training that is required to operate such systems and to optimize its image quality, saving resources and enabling operation in underserved regions.

Consequently, to meet the evolving demand for high‐quality point‐of‐care low‐field imaging, this work introduces the Nexus console, a versatile and performant MRI console, which can incorporate a variety of different sensor information via multiple receive channels. The console serves as a control interface and as a high‐performance reconstruction system, enabling real‐time data processing steps. By harnessing the capabilities of *Python*‐based open‐source software and adhering to established, open‐source MRI sequence description standards^37^ and open data formats,^39^ the console facilitates seamless integration into existing MRI workflows while offering unprecedented customization options and fast prototyping. The open‐source design not only enables full control and understanding of the forward model, but also democratizes the access to MRI by lowering the costs of high‐end components, which are required to implement robust imaging.

In this manuscript, the hardware and software design of the Nexus console^42^ is presented, highlighting its key features and capabilities. Although the console is suitable for a range of frequencies up to about 15 MHz (B_0_ ˜ 0.35 T) in its current configuration, in this work we demonstrate the application in low‐field imaging at B_0_ ˜ 50 mT (i.e., on the portable, open‐source, low‐field MRI scanner OSI^2^ ONE).^24^ Demonstrations include three‐dimensional (3D) in vivo imaging, EMI detection, and mitigation using external sensors, temperature monitoring, B_0_ field monitoring, and self‐monitoring of MR hardware components to perform system calibrations and adjustments. With these advances, we aim to enhance the capabilities of low‐field MRI systems, improving image quality and enabling robust operation and reproducibility of results. All content in this manuscript is shared open source, including the hardware components, software for operating the console, and all sequences and postprocessing algorithms, available at www.opensourceimaging.org/project/nexus‐console/.

## 2 METHODS

### 2.1 Hardware architecture

**FIGURE 1:**
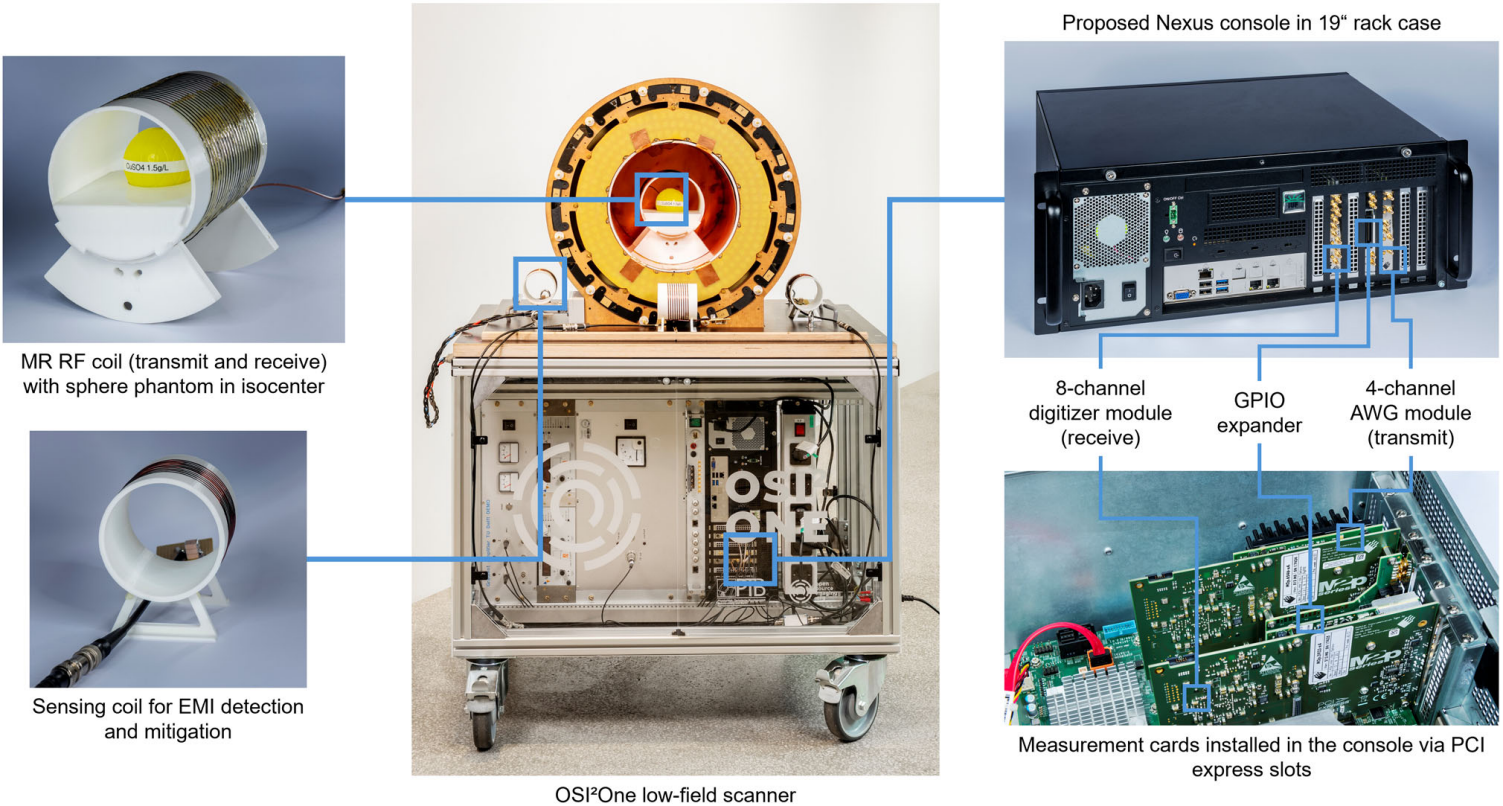
Overview of the experimental setup with electromagnetic interference (EMI) detection and mitigation using the Nexus console within the portable OSI^2^ ONE low‐field MRI scanner, which is very similar to the setup used for in vivo experiments. Three EMI‐sensing coils, tuned to the Larmor frequency, are positioned around the system, either in parallel or orthogonal to the MR coil. The Nexus console uses an eight‐channel digitizer (receive) and four‐channel arbitrary waveform generator (AWG) (transmit) module from Spectrum Instrumentation, shown on the right side.

The console is inspired by concepts described by Winter et al.^43^ and is based on a X12SPL‐F motherboard (Supermicro Computer Inc., San Jose, CA, USA) with a 24‐core 6312U CPU (Intel Corporation, Santa Clara, CA, USA) and 256 GB of RAM, housed in a 19‐in. rack case (Figure 1). It includes seven PCI Express slots, which are used for measurement cards (Spectrum Instrumentation GmbH, Großhansdorf, Germany).

The digital‐to‐analog converter is a 16‐bit arbitrary waveform generator (AWG) module (M2p.6546‐x4), providing four analog transmit channels at up to 40 MS/s and ±12 V output. It includes 512 MS on‐board memory and four GPIO ports, expandable to 20. The analog‐to‐digital converter (ADC) is the 16‐bit digitizer module M2p.5933‐x4, with 40 MS/s sampling on up to eight single‐ended or four differential receive channels, with 512 MS on‐board memory and 700 MB/s streaming capability. The cards allow direct data streams to or from CUDA GPUs and synchronize with up to 15 cards, if extra channels are required. The installed measurement cards can operate MR systems up to about 20 MHz, while upgrading to higher‐performance cards enables the operation of systems at even higher field strengths.

**FIGURE 2:**
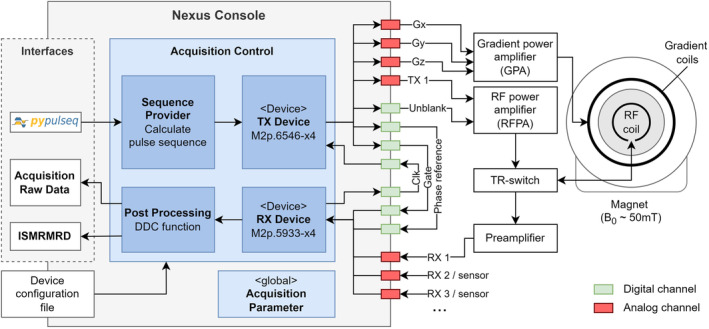
Overview of the Nexus console system setup. The MRI acquisition is controlled by a *Python*‐based software architecture to play out the pulse sequence and to measure the MR signal. The arbitrary waveform generator (AWG) modules generate gradient (Gx, Gy, Gz) and radiofrequency (RF) transmit (TX 1) signals which are amplified by gradient power amplifier (GPA) and radiofrequency power amplifier (RFPA). The received signal (RX 1) from the transmit‐receive (TR)‐switch is pre‐amplified and measured by the digitizer module. The interface to the Nexus console is defined by community‐developed standards, including *PyPulseq* and ISMRM raw data (ISMRMRD).

Figure 2 shows the system and console architecture used for the OSI^2^ ONE low‐field MRI system (Larmor frequency f_0_ ˜ 2 MHz).^24^ The RF transmit signal is transferred directly to an RF power amplifier (RFPA), which routes the amplified RF pulses via a passive transmit/receive switch to a single‐channel RF coil. The three gradient waveforms (Gx, Gy, Gz) from the AWG module directly connect to the gradient power amplifier (GPA). The received MR signal is pre‐amplified by a ABL0050‐00‐4510 preamplifier (Wenteq Microwave Corporation, Monrovia, CA, USA) before being sampled by one of the receive channels (RX1) of the digitizer module. Two GPIO ports are configured for unblanking of the RFPA and gating the digitizer module. To ensure precise timing and synchronization, clock and phase reference signals are transferred by two additional GPIO ports.

For this study, we evaluated the console at two different sites at Leiden University Medical Center (LUMC) and Physikalisch‐Technische Bundesanstalt (PTB) Berlin. The PTB setup includes a 1‐kW open‐source RFPA^44^ and GPA.^45^ LUMC uses a 250‐W RFPA (Barthel HF‐Technik, Aachen, Germany) and a B‐AFPA 40 GPA (Bruker Corporation, Billerica, MA, USA).

### 2.2 Software architecture

#### 2.2.1 System environment

At PTB, we use *AlmaLinux* version 9.3 as the operating system for the console computer. The spectrum cards are operated with the kernel version M2p 2.17 build 17916 and library version 6.05 build 21120. At LUMC, the console is implemented with *Microsoft Windows 10* as the operating system.

The console software, implemented in *Python*, includes key modules highlighted in blue in Figure 2: acquisition control, sequence provider, transmit, and receive devices to conduct the MR experiments. On startup, instances of these classes are created from configuration file parameters. The acquisition control instance manages all the other instances, enabling script‐based MR experiments and serving as a backend for user interfaces. The software and measurement‐card driver interface are implemented in *Python*, allowing easy customization.

#### 2.2.2 Execution of an MR pulse sequence

The console software interfaces directly with Pulseq^37^ sequences using the *PyPulseq* ^38^ implementation. Pulseq sequences are divided into blocks, each containing up to three gradient events, an RF event, and an ADC event. The sequence provider in Figure 2 extends the *PyPulseq* sequence class, introducing methods for loading, calculating, and visualizing the sequence.

During sequence calculation, gradient events are interpolated and written to pre‐allocated Numpy arrays. Pulseq defines gradients in kHz/m; these are scaled to mV by the gradient coil efficiency, GPA gain, gyromagnetic ratio, and a scaling factor for the field of view (FOV). The resulting gradient waveforms are stored as int16 values for interpretation by the spectrum‐card AWG. Gradient waveform resolution depends on the AWG card hardware and can be set per channel. RF pulse envelopes are similarly interpolated, scaled from Hz to mV using a B_1_‐scaling factor, and digitally modulated with the Larmor frequency. Both scaling terms are defined in the acquisition parameters and can be determined by calibration protocols.

Digital control signals, including the RFPA enable signal, ADC gate, and phase reference signal, are transmitted synchronously with the analog waveforms. The 16th bit of the gradient waveforms stores these digital signals, reducing gradient channel precision from 16 to 15 bits, what is also commonly used for commercial MRI systems. However, due to the high degree of oversampling, a significantly higher effective resolution can be achieved if easy‐to‐implement dither methods are used. Gradient and RF waveforms are stored block‐wise in an int16 Numpy array using Fortran order and concatenated into a *Python* list object.

Each sequence calculation is specific to an acquisition parameter definition, which includes Larmor frequency, B_1_ scaling, FOV scaling, and gradient offset. These parameters are gathered in a unique, hashable dataclass, allowing validation of calculated sequences before execution.

**FIGURE 3:**
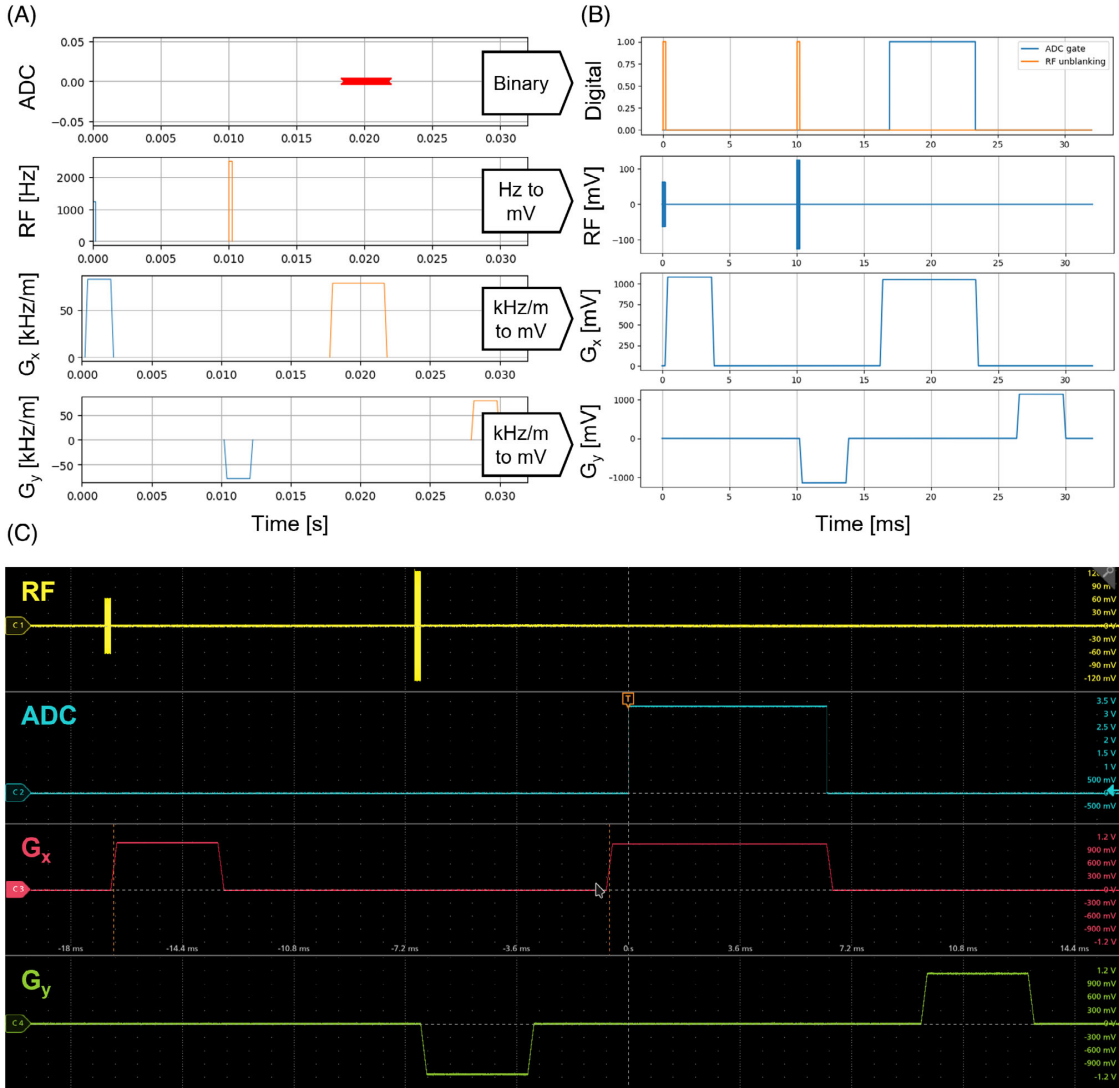
Comparison of calculated pulse sequence using *PyPulseq* (A), unrolled sequence including radiofrequency (RF) transmit waveform, gradient (Gx, Gy) waveform and analog‐to‐digital converter (ADC) gate to be played out (B), and measured waveforms at the Nexus console output on a digital oscilloscope (C).

Figure 3 shows a section of a two‐dimensional spin‐echo pulse sequence written in *PyPulseq*, calculated on the console and measured by an oscilloscope.

#### 2.2.3 MR signal acquisition

The transmission card is controlled by a TxDevice class instance, and the digitizer card is controlled by a RxDevice class instance. Both classes are inherited from a common abstract device class, which implements methods that both classes have in common. The TxDevice and RxDevice operation loops are both managed by the acquisition control and run in concurrent *Python* threads.

When playing out a sequence, first the AWG card buffer is filled with the calculated sequence samples. During sequence execution, the AWG card buffer operates as a ring buffer. Until the last sequence sample has been transferred, the TxDevice operation loop continuously reads the available memory size of the AWG card, which is updated whenever memory has become available in the amount of a notify size. As soon as new available memory is released, the next part of the sequence is transferred to the AWG card memory; thereby, the sequence is streamed to the AWG card until every sample point has been transferred or until the operation loop is interrupted by the acquisition control.

The digitizer card, controlled by the RxDevice class, is operated in gated timestamp mode. The card features a sample point buffer and a separate timestamp buffer, which is continuously monitored within the operation loop. The registration of two timestamps in the timestamp buffer means that an ADC event is complete and the memory transfer from the digitizer card to the computer memory is started. By the timestamp difference, the ADC duration and subsequently the number of ADC sample points are calculated. After the corresponding data have been received and written to card memory, the timestamp buffer is reset, and the received sample points are transferred to one int16 Numpy array in the main memory. The array is organized in Fortran order, as the data from all the active channels are received in parallel. These data are restructured to have the channels in the first and the readout samples in the second dimension, before being added to a list of acquisition events. As the memory transfer only started as soon as the second timestamp has also been detected, the total number of ADC sample points for all active channels is currently bounded by the on‐board card memory of 512 MS. Once started, the receive loop runs continuously until the thread is interrupted. Possible interruption sources are the completeness of expected ADC gates, error during sequence execution, or a sequence execution timeout, which is given by the sequence duration and an additional buffer time.

#### 2.2.4 Postprocessing

The acquisition control class handles postprocessing immediately after acquisition. ADC gates are first concatenated into a Numpy array with dimension averages, coils, gates/phase encodings, and readout samples. The phase reference signal, encoded in the 16th bit of Channel 1, is then separated from the analog signals, which are converted to floating‐point values in mV. Demodulation corrects the phase information of both analog and digital phase signals, followed by decimation using the SciPy finite impulse response filter.^46^ The decimation factor is determined by the receive bandwidth. The entire workflow is digital, providing access to unprocessed “raw” data.

Processed and raw data, along with the sequence, acquisition parameters, device configuration, and experiment metadata, are returned in an acquisition data object. When writing to main memory, data are stored in a Numpy array and can additionally be exported to ISMRM raw data (ISMRMRD)^39^ format. The standardized ISMRMRD format ensures compatibility with existing image‐reconstruction frameworks.^40^ Storing raw data is optional due to its large memory requirement. The sequence is stored in a Pulseq file, and all parameters and metadata in a joint JSON file.

### 2.3 Reproducibility

To replicate the console system, the initial step is the assembly of the computer system, which requires moderate understanding of computer hardware. All components are modular, and the measurement cards can be inserted easily into the PCI Express slots on the motherboard. The entire setup requires less than 8 person‐hours and only a screwdriver, with no additional machines or tools needed.

It is essential to install the driver for the measurement cards by following the instructions provided by Spectrum Instrumentation. Note that this part depends on the operating system of the console. The manual includes critical guidelines for the assembly of the measurement cards. Last but not least, the Nexus‐console *Python* package, available on https://github.com/schote/nexus‐console under the GPL‐3.0 open‐source license, must be installed, preferably within a virtual environment to ensure proper isolation and ease of management. The computer system with the measurement cards described in Section 2.1 costs less than 12 000 €, which corresponds to one‐fifth the cost of a comparable commercial system with proprietary software. Lower cost alternatives are based on software‐defined radios with dedicated open‐source software frameworks at material costs of 1000–2000 €.^41^ However, they lack built‐in computational power, have memory and performance limitations (e.g., fewer transmit and receive channels), and demand significantly more labor, knowledge, and skill for assembly.

### 2.4 Experimental setup

#### 2.4.1 System configuration

The console was tested on two different OSI^2^ ONE scanners at the PTB in Berlin, Germany, and the LUMC in Leiden, Netherlands, using two different RFPAs and two different GPAs described previously. The gradient channels were configured to maximum output amplitude to cover the full range of the GPAs. The RF output channel was configured to provide a maximum output of 200 mV. The sampling rates of the AWG and the digitizer module were 20 MS/s. This resulted in an oversampling factor of 10 for the digitally modulated RF waveform with a sample point spacing of 50 ns. With the current implementation, the maximum ADC duration was 25.6 s with one receive channel and 3.2 s with eight receive channels, much higher than practical ADC durations of up to 10 ms.

#### 2.4.2 In vivo imaging

To evaluate the performance of the console, in vivo imaging experiments were performed with one healthy volunteer giving informed consent according to the policies of the LUMC ethics committee. A 3D turbo spin‐echo (TSE) sequence using Cartesian sampling with two phase‐encoding (PE1 and PE2) and one readout gradient has been implemented in *PyPulseq*. To obtain images with proton density (PD)–weighted contrast, an inside‐out trajectory with echo time (TE)/repetition time (TR) of 20/500 ms and an echo train length (ETL) of 5 was used. For T_2_ contrast, a linear trajectory with TE/TR of 20/2000 ms and an ETL of 22 was applied, resulting in an effective TE of 220 ms. Both contrasts of the 3D whole‐brain images were acquired with a FOV of 240 × 200 × 210 mm (RO, PE1, PE2) and a resolution of 2 × 2 × 5 mm (RO, PE1, PE2). For excitation and refocusing, a 200‐μs block RF pulse was used. Three dummy shots were applied before the imaging sequence to reduce saturation effects. The readout bandwidth for both contrasts was set to 20 kHz, which resulted in an ADC duration of 6 ms with 120 readout samples and a finite impulse response decimation factor of 1000. A fast Fourier transform was used to reconstruct the image.

Before imaging, calibration scripts based on an FID sequence were executed using the console to determine the exact Larmor frequency, power for a 90º tip angle, and B_0_ shimming (first order using the three gradient coils). For active B_0_ shimming, we calibrated gradient offset values, which were applied to each of the gradient coils to obtain static linear shimming. Due to the presence of eddy currents, the refocused MR signal might not exactly occur at the center of k‐space during readout. To compensate for this effect, a gradient correction time of 160 μs was set, which was added to the readout gradient flat time before opening the ADC gate. The gradient correction time was expected to be independent of the subject and was determined previously from a spherical phantom. To prevent electromagnetic interferences, a Faraday cage was placed over the subject.

#### 2.4.3 Integration of external sensor information

To demonstrate the advantages of having additional receive channels available for the console, several system parameters were recorded during acquisition that could be used to calibrate the system. On the receive side, these channels monitored the main RF coil, pickup RF coil, forwarded power of the RFPA, reflected power of the RFPA, and the GPA monitor for each of the x‐, y‐, and z‐gradient channels.

For this reason, a short monitoring sequence was designed using the *PyPulseq* framework. The monitoring sequence consisted of a 400‐μs RF block pulse, followed by three consecutive trapezoidal gradients with an amplitude of 80 kHz/m and a duration of 2 ms for Channels x, y, and z. The spacing, as well as the delay at the beginning and the dead‐time at the end of the sequence, were set to 1 ms. The ADC gate was configured to cover the entire sequence, starting at the end of the delay and ending at the beginning of the dead‐time. This sequence visualized, for example, coil ringing, and the isolation effectiveness of the transmit/receive switch. Regarding the gradient waveforms, the monitoring sequence allowed for fine‐tuning of the GPA gain factor to address any impedance mismatch between the console and GPA. It could also be used to estimate a timing correction factor for the readout gradient.

#### 2.4.4 EMI detection and mitigation

To demonstrate the versatility of the console, we integrated the low‐field MRI system with three EMI sensing coils and acquired images on a simple phantom. We generate an artificial interference signal in the range of the readout bandwidth by an external antenna to emulate the feasibility of EMI detection and removal using the console. For image acquisition, a two‐dimensional version of the TSE sequence was used, with TR/TE of 600/14 ms, ETL of 18, isotropic FOV of 150 mm, readout and phase‐encode dimensions of 120/120, and a readout bandwidth of 20 kHz, leading to a total acquisition duration of 4.2 s. The MR coil was a solenoid coil with 140 mm in diameter and a spherical phantom with 7 cm in diameter positioned in the isocenter. Each of the EMI sensing coils had 10 turns, measured 80 mm in diameter, and was tuned to the Larmor frequency of the system. We used a ZFL‐500LN preamplifier (Mini‐Circuits, Brooklyn, NY, USA) with 24/2.9‐dB gain/noise figure for each of the EMI coils. All the coils were positioned next to the Halbach array: two in parallel to the bore and one rotated 90º, as shown in Figure 1. On the console, we enabled four receive channels to acquire the MR signal and the three EMI coils synchronously. The EMI‐mitigated image was computed using a *Python* implementation of EDITER.^36^

#### 2.4.5 Temperature and B_0_ field measurement

Using seven fiber Bragg grating optic temperature sensors (imc Test & Measurement GmbH, Berlin, Germany) positioned in the interspaces of the permanent magnets and inside the magnets bore as depicted in Figure 9A, temperature was monitored over different stages of an MRI examination. In parallel to temperature assessment, the static magnetic B_0_ field was monitored inside the bore using the PT2026 NMR probe (Metrolab, Plan‐les‐Ouates, Switzerland). At the beginning of the first stage, the system was turned on, and after approximately 20 min, the subject was positioned in the scanner. After approximately 10 min, the 3D T_2_‐weighted TSE sequence was executed, whereafter the subject was taken out of the scanner and another 20 min at rest were recorded. The timestamp‐based measurement data from the additional measurement hardware were accumulated by the console and retrospectively synchronized.

## 3 RESULTS

### 3.1 In vivo imaging

**FIGURE 4:**
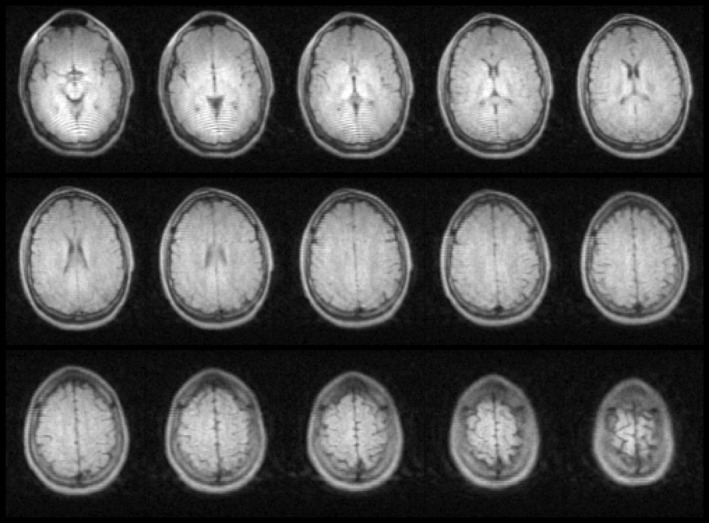
Proton density–weighted in vivo brain image contrast using the Nexus console on an OSI^2^ ONE v1 scanner. Displayed are 15 slices from a three‐dimensional turbo spin‐echo sequence with echo time/repetition time of 20/500 ms, field of view of 200 × 210 × 200 mm, and resolution of 2 × 2 × 5 mm. The total image acquisition time was 5 min 52 s.

**FIGURE 5:**
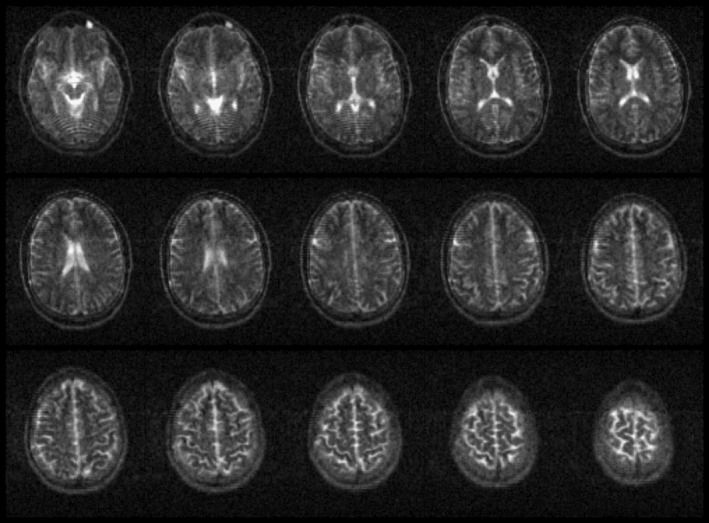
T_2_‐weighted in vivo brain image contrast using the Nexus console on an OSI^2^ ONE v1 scanner. Displayed are 15 slices from a three‐dimensional turbo spin‐echo sequence with echo time/repetition time of 20/2000 ms, field of view of 200 × 210 × 200 mm, and resolution of 2 × 2 × 5 mm. The total image acquisition time was 6 min 28 s.

PD‐weighted and T_2_‐weighted 3D data sets of a healthy volunteer's brain acquired with the Nexus console are displayed in Figures 4 and 5, respectively. The in‐plane pixel resolution in each image is 2 × 2 mm with a through‐plane resolution of 5 mm. Fifteen of a total of 42 slices are shown in an axial orientation. The acquisition durations are 5 min 52 s and 6 min 28 s, respectively.

**FIGURE 6:**
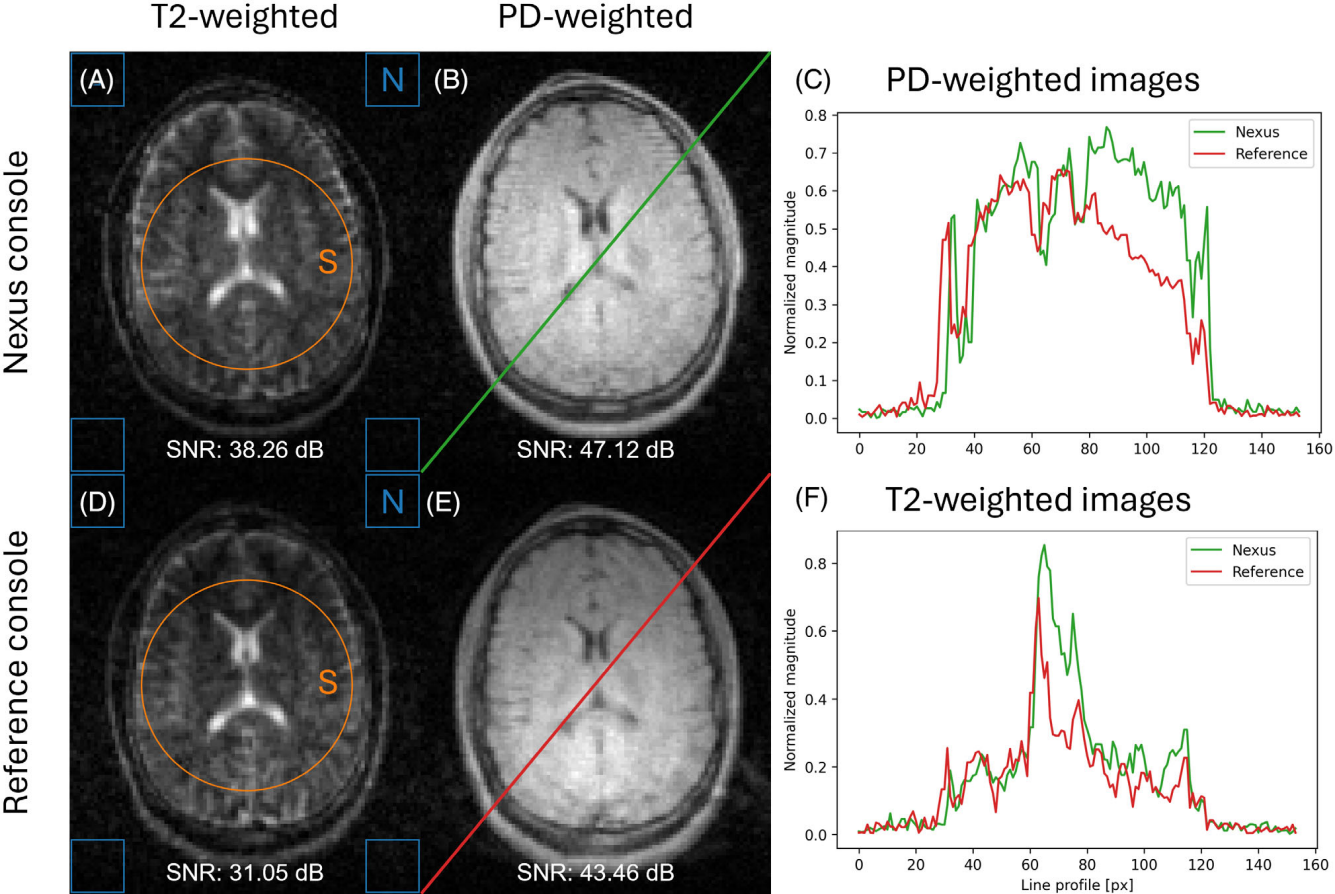
Comparison of proton density (PD)–weighted and T_2_‐weighted image contrasts obtained from a three‐dimensional turbo spin‐echo sequence with the proposed Nexus console (A,B) and a commercially available reference console (Kea2) (D,E). The ratio of signal (orange area in [A] and [D]) to noise (blue squares in the corner of [A] and [D]) is denoted as signal‐to‐noise ratio (SNR) at the bottom of each image. (C) Comparison of the intensity profiles along the diagonals in (B) and (E) of the magnitude images with PD weighting. (F) The same comparison for the T_2_‐weighted images of the Nexus console and the reference.

As a reference comparison, we show images in Figure 6 acquired with the same imaging protocols using a Kea2 spectrometer (Magritek Inc., Aachen, Germany), which has been used previously for imaging on the low‐field MR system.^5^ Because the reference console is not Pulseq compatible, the 3D‐TSE sequence with identical parameters was implemented in the native console software. Differences in the Kea2 architecture compared with the Nexus console are the use of a combined cascaded‐integrator‐comb and finite impulse response filter, an integrated pre‐amplifier with a gain/noise figure of 36/1.2 dB, a second‐stage variable‐gain pre‐amplifier (used at 22 dB gain), a passive transmit/receive switch, and an anti‐aliasing filter with 40‐MHz cutoff frequency. k‐Space data were first exported from the console software environment and reconstructed in *Python*. For both consoles, the 90° RF pulse amplitude was calibrated to the maximum FID signal integral.

Figure 6 illustrates PD‐weighted and T_2_‐weighted images from the Nexus console and the Kea2. The experiments with the Nexus console and the Kea2 were carried out on consecutive days, so there were slight variations in the positioning of the subject. The same RF coil, RFPA, and GPA were used for both imaging sessions. An estimation of SNR as shown in Figure 6 according to NEMA^47^ yielded about 8.4% and 23.2% improvement for PD and T_2_ contrast using the Nexus console. The PD‐weighted and T_2_‐weighted images from our proposed console showed a zebra artifact along the readout dimension in the upper part of the brain. This is caused by miscalibration, resulting in signal leaking into the outer k‐space regions. The Kea2, which underwent individual calibration, did not show such artifact, but the images exhibited a decrease in intensity that was particularly visible in the profile comparison of Figure 6.

The Nexus console supports the export of the acquisition data in ISMRMRD format, which enables reconstructions with existing frameworks such as Gadgetron.^40^ For the image reconstruction, a docker container running the Gadgetron client was used to carry out image reconstruction using fast Fourier transform and to save the obtained images in DICOM format (see S1).

### 3.2 Monitoring

**FIGURE 7:**
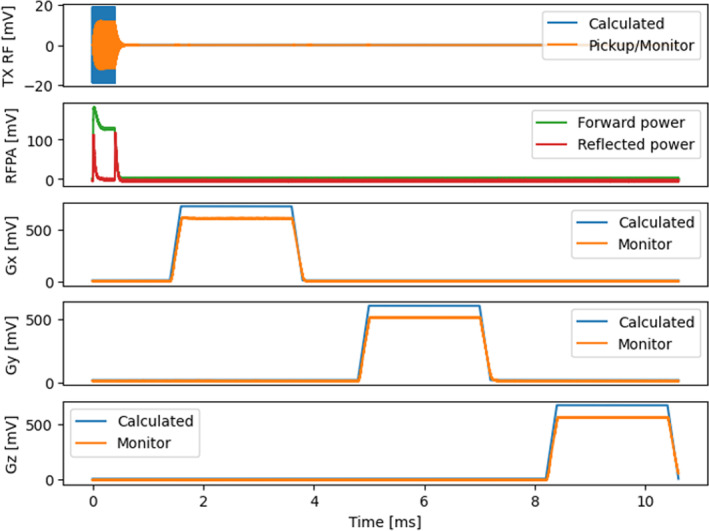
Monitoring of radiofrequency power amplifier (RFPA) and gradient power amplifier using the Nexus console. The first row shows the calculated radiofrequency (RF) waveform (*blue*), and the signal measured at the pickup coil (*orange*). The reflected and forward power monitor of the RFPA is plotted in green and red in the second row. All the gradient channels are shown from Rows 3 to 5, where blue represents the calculated waveform and orange the measured monitor signal.

Using the additional input channels of the Nexus console, the measured RF performance and the GPA monitor compared with the calculated values are shown in Figure 7. This allowed easy detection of the resulting RF envelope, the forward and reflected power, and the gradient monitor. To plot the measured signals against the calculated waveforms, we used the unprocessed signal, sampled at 20 MS/s. From the pickup coil signal, we could identify clear deviation from the ideal RF waveform, which correlated to the spikes in the RFPA monitor signals. Similarly, as expected, the measured gradient signals showed a slight delay in comparison to the calculated waveforms. To cover the full range of the Nexus consoles analog outputs, a high impedance was required at the GPA inputs. The deviation between monitored and calculated gradient waveform can be explained by the GPA input impedance being too low. The deviation of the resulting FOV can be compensated using the monitoring signal; however, this also reduces the maximum console output.

### 3.3 EMI detection and mitigation

**FIGURE 8:**
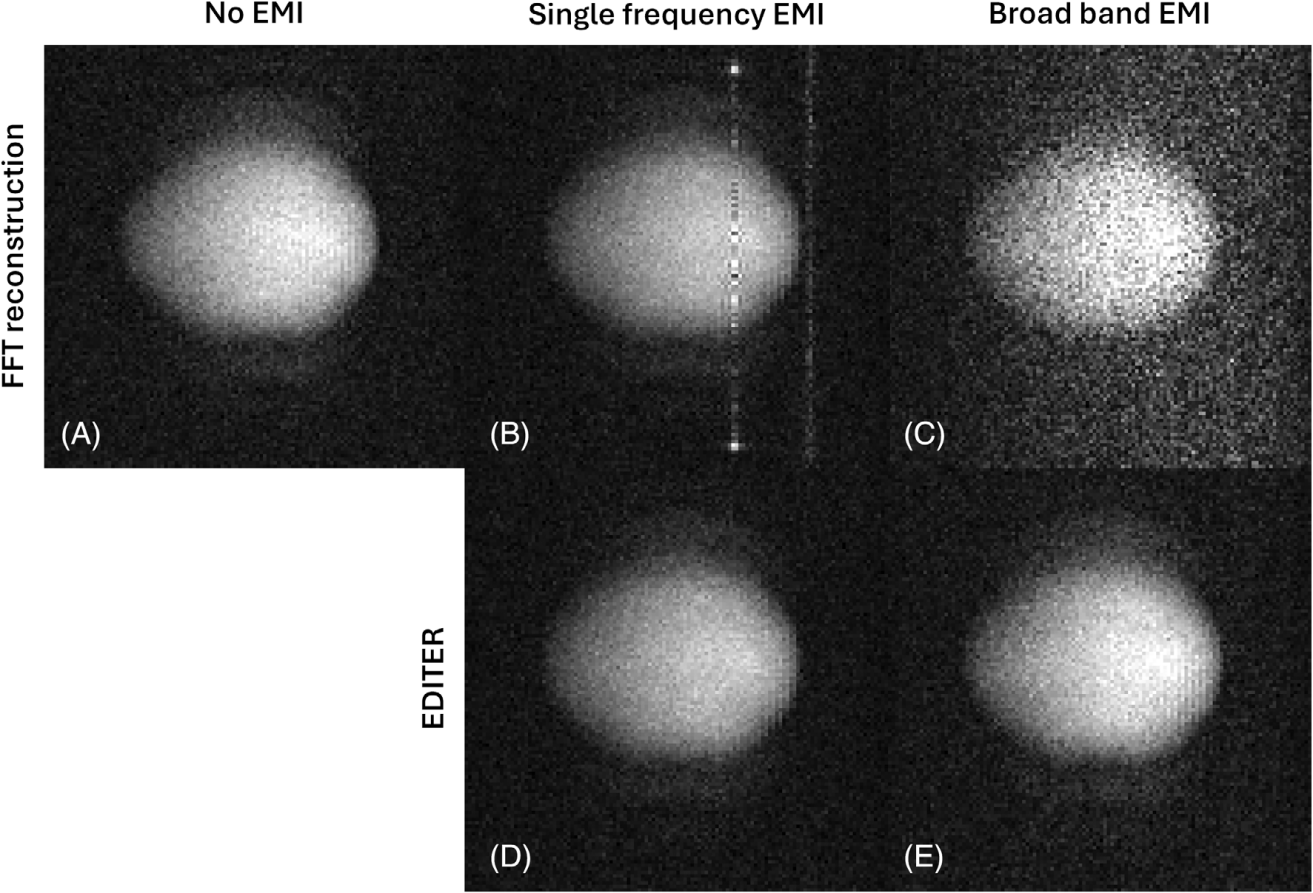
Electromagnetic interference (EMI) detection using additional receive channels of the Nexus console with the system installed at Physikalisch‐Technische Bundesanstalt and mitigation using the EDITER algorithm. Three EMI‐sensing coils were positioned parallel, orthogonal, and 45º rotated to the system's bore opening. A sphere phantom of approximately 7 cm in diameter was imaged using a two‐dimensional turbo spin‐echo sequence (echo train length of 18). Image reconstruction using fast Fourer Transform without EMI (A), with single‐frequency EMI showing zipper (B) and with broadband EMI showing decreased signal‐to‐noise ratio (C). Mitigated EMI using EDITER (D, E).

The results of the EMI detection by three sensing coils and its suppression using *EDITER* are displayed in Figure 8. The image without any artificial interference using an RF shield is shown in Figure 8A. Two examples of EMI are shown: a signal with a constant frequency in Figure 8B and with a broad frequency band of 100 kHz in Figure 8C. In both cases, the interference was clearly visible. Using the *EDITER* algorithm, the EMI artifact could be successfully mitigated, as shown in Figure 8C,D. A more inhomogeneous B_0_ field (> 4000 ppm) in this experimental setup, compared with the in vivo setup, causes the geometric distortions of the sphere phantom.

### 3.4 Temperature and B_0_ field monitoring

**FIGURE 9:**
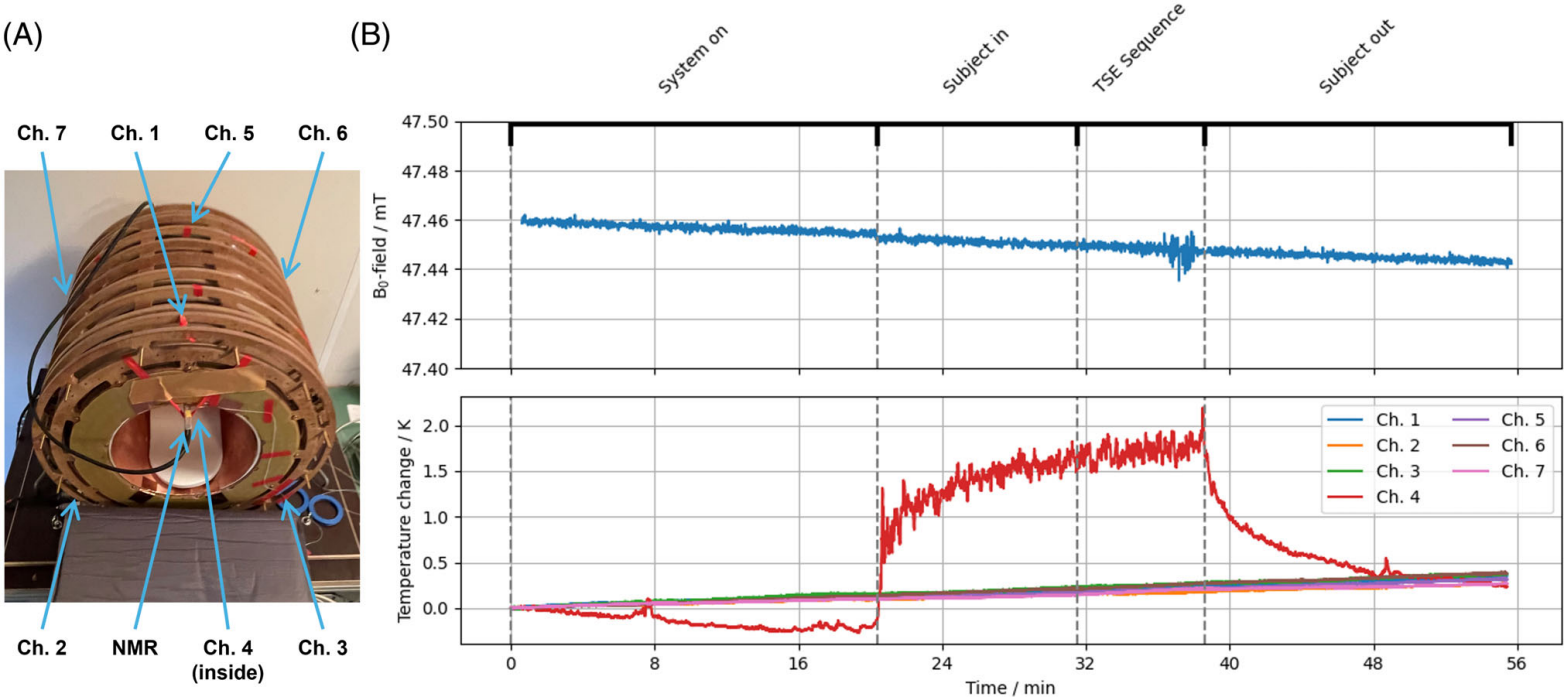
(A) Experimental setup. (B) Measurement results for temperature and B_0_ field assessment during an MRI examination. Seven fiber Bragg grating (FBG) temperature sensors and an NMR probe were set up at the system. The experiment investigated temperature changes during system on‐time, with a subject in the scanner and during imaging, and their effect on the B_0_ field. Approximate time periods are marked with dashed vertical lines in (B). The temperature increase, measured at Channel 4 inside the bore, does not affect the B_0_ field. During turbo spin‐echo (TSE) sequence execution (˜7 min), the increasing temperature causes a B_0_ field drift of about 2.63 μT, which corresponds to about 112 Hz.

Figure 9B shows the monitoring results of the B_0_ field and the temperature at seven different positions in between the permanent magnets and inside the magnet bore. The temperature measured in between the permanent magnets increased by less than 0.5 K during the examination, most likely due to an increase in the room temperature. The rise in temperature correlated with a B_0_ field decrease by 20 μT but was independent of the subject or pulse sequence. The B_0_ field shift during the 3D‐TSE pulse sequence in Stage 3 was about 2.63 μT, which corresponds to about 112 Hz. The increase of approximately 2 K of Probe 4 can be explained by airflow through the subject's respiration. However, this does not influence the mean B_0_ field, except for a small drop at the beginning of Stage 2 due to a change in the magnets load.

## 4 DISCUSSION

In this study, the new versatile Nexus console for advanced low‐field MRI has been designed and demonstrated. The implementation is based on an open‐source framework, allowing easy access and customizability. The demonstrated setup has four transmit and eight receive channels that can easily be extended by additional measurement cards. In our current setup, there are seven PCI Express slots on the mainboard, technically allowing five more cards (four to eight channel per card) to be additionally embedded in the console, leading to a total number of 32–52 channels depending on the configuration. Because the cards themselves allow for synchronization of up to 16 measurements cards, even higher channel numbers are possible in a different hardware configuration.

Without any modification of the imaging protocol, we set up the console with three (up to seven) additional EMI‐sensing coils and corrected for different EMI scenarios using the EDITER^36^ algorithm. To emphasize versatility, we monitored temperature and B_0_ field over an MRI examination with additional measurement hardware. By synchronizing the readout of such sensors, Larmor frequency can be tracked automatically in the future. The receive and transmit channels are not limited to these examples but can accommodate a multitude of different auxiliary sensors such as additional B_0_ field probes and current drivers for B_0_ shim coils^33^ to improve B_0_ field homogeneity under varying environmental conditions, sensors to detect and correct motion artifacts,^48^ physiological monitoring devices such as electroencephalography^49^ or electrocardiography,^50^ sensors for RF pulse monitoring or gradient field monitoring,^51^ or additional RF receive channels for parallel imaging.^52^

Implementation in *Python* and the native support of *PyPulseq* sequences allowed seamless development and execution of TSE‐based sequences for 3D in vivo imaging. PD and T_2_‐weighted in vivo images showed comparable image quality to a commercially available state‐of‐the‐art console. An increased SNR with the Nexus console was observed, which could be due to the differences in the receive chain, postprocessing, or from environmental differences between the experiment days. In contrast to the proprietary reference, the fully digital realization of the Nexus console and the transparent open‐source software provided full access to the unprocessed raw sample points before down conversion, and full control of the data processing pipeline. Currently, the calculation of the waveforms from the Pulseq sequence is performed before the acquisition. This causes a deadtime for the start of the sequence. However, by using parallel computing, the sequence calculation can be shortened and even performed at runtime in parallel to the execution of the sequence. Alternatively, one can also set up different transmit cards with different sampling rates for RF and gradient waveforms or output the gradient waveforms by the digital channels using a slower external digital‐to‐analog converter, such as the OCRA1^53^ board. The ISMRMRD interface enables existing frameworks, such as Gadgetron,^40^ for image reconstruction. Having the option to directly streamline data samples to a GPU also enables artificial intelligence–based reconstructions and decrease the overall computation time.

Within the absolute limits (40 MS/s) of the measurement cards used in the Nexus console, the sampling rate is freely configurable to adjust the experimental setup to a target frequency range. According to Nyquist MR experiments using the console with less than 20 MHz (e.g., 15 MHz) (B_0_ ˜ 0.35 T) would be feasible. Direct access to the unprocessed raw data samples at up to 40 MS/s allows us to investigate oversampling techniques that can be used to boost the SNR.^54^ If higher frequencies are required, AWG and digitizer cards with higher sampling rates could be used, which are compatible with only minor modifications to the current open‐source software framework. This has been tested at 7T frequencies (f_0_ ˜ 300 MHz) using sampling rates of 1.25 GS/s and an eight‐channel parallel‐transmission medical implant safety testbed.^43^

With a total material cost of about 12.000 € and no need for specialized skills or tools, the Nexus console is easily reproducible and cost‐effective, given its performance. It bridges the gap between existing low‐cost open‐source designs, which are underpowered for advanced applications, and expensive proprietary systems. Assembly is as straightforward as building a PC, using standardized computer hardware housed in a 19‐in. enclosure. This simplicity enhances reproducibility for low‐field MRI research and facilitates standard conformity procedures for electrical safety in compliance with IEC 60601‐1,^55^ easing approval for in vivo use. The console's measurement cards, already used in a medically approved MRI scanner,^56^ are currently being integrated into the open‐source OSI^2^ ONE scanner^24^ within the A4IM^57^ and OpenLab MedTech^58^ projects, where documentation will be released that aligns mostly with the EU regulation on medical devices MDR (EU)2017/745. This will simplify technology transfer from scientific instruments to products and knowledge transfer from high to low resource settings. The Nexus console combines an open‐source framework with powerful hardware to deliver advanced low‐field MRI capabilities for a clinical setting, reducing the barriers to access and supporting the democratization of MRI, particularly in underserved regions.

## 5 CONCLUSION

Nexus, a new versatile console for low‐field MRI, is proposed, which consists of open‐source software and a setup accommodating many transmission and reception channels. This configuration facilitates easy integration of a multitude of different auxiliary sensors for EMI mitigation, system monitoring, and calibration. The additional information allows fast and more complex image reconstruction and real‐time feedback mechanism.

## Supporting information


**Figure S1.** Demonstration of a Gadgetron^27^‐based image reconstruction using the data acquired with the Nexus console. (A,B) Image slices with proton density (PD)–weighted (A) and T_2_‐weighted (B) contrast. The acquisition raw data were exported in ISMRM raw data (ISMRMRD) format and reconstructed to a DICOM image using the Gadgetron toolbox. The image slices were exported from a DICOM viewer.

## Data Availability

The Nexus console software code used for this study is publicly available on GitHub at https://github.com/schote/nexus‐console and is provided under the terms of the GPL‐3.0 license. The specific version of the code associated with this publication is referenced by the commit SHA ff983c99099de5527431167bd2ef97efd03ea444.
