# Supplementary material for: Nexus: A versatile console for advanced low‐field MRI
Source: Magn Reson Med. 2025 Jan 27;93(5):2224–38. doi: 10.1002/mrm.30406 (PMC11893031; doi:10.1002/mrm.30406)
Supplement: Supplementary file 1 — Figure S1. Demonstration of a Gadgetron 27 ‐based image reconstruction using the data acquired with the Nexus console. (A,B) Image slices with proton density (PD)–weighted (A) and T2‐weighted (B) contrast. The acquisition raw data were exported in ISMRM raw data (ISMRMRD) format and reconstructed to a DICOM image using the Gadgetron toolbox. The image slices were exported from a DICOM viewer. [file MRM-93-2224-s001.pdf]

Supplementary Material

S1. Supplementary Figure: Reconstruction with Gadgetron

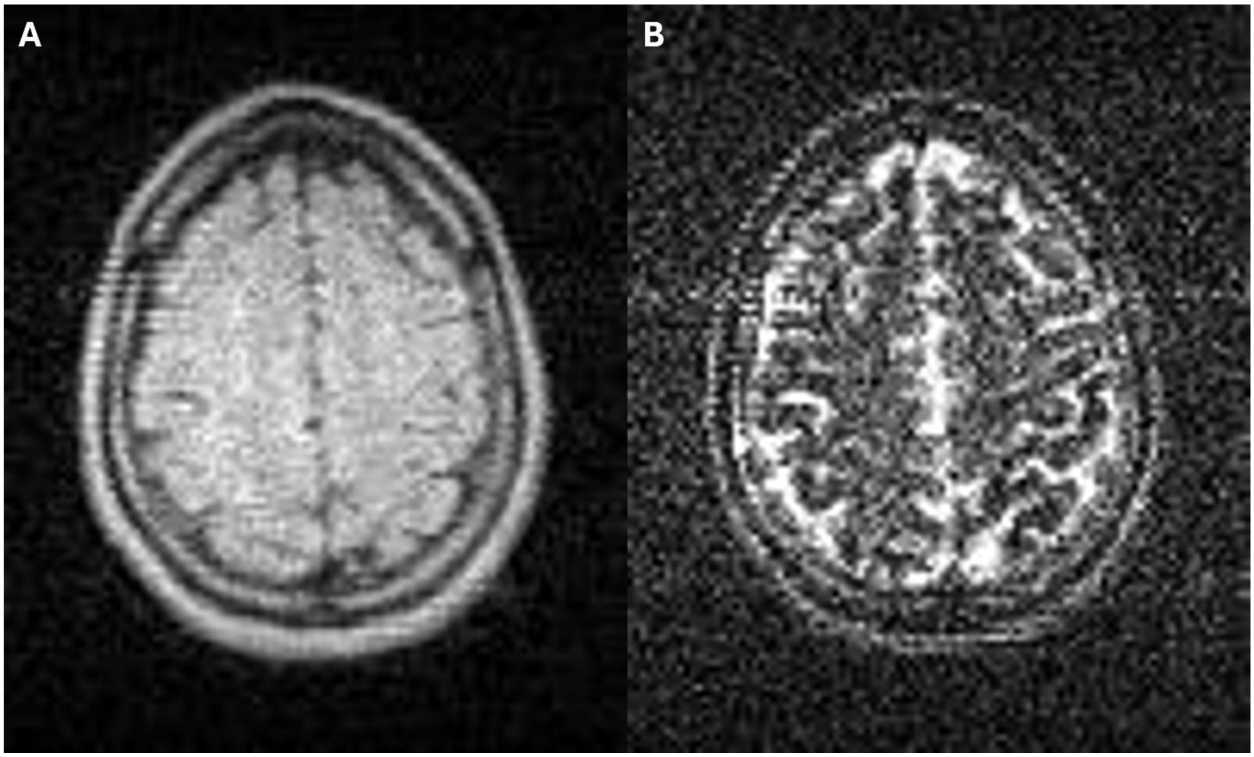

Figure 10 Demonstration of a Gadgetron[28] based image reconstruction based on the data acquired with the Nexus console. Image slices with PD weighted (A) and T_2 weighted (B) contrast are shown. The acquisition raw data has been exported in ISMRMRD format and reconstructed to a DICOM image using the Gadgetron toolbox. The image slices were exported from a DICOM viewer.
